# Comprehensive Evaluation of the Performance and Benefits of SSA–GGBS Geopolymer Mortar

**DOI:** 10.3390/ma16114137

**Published:** 2023-06-01

**Authors:** Tao Zhang, Xiaoshuang Shi, Qingyuan Wang, Wenbin Peng

**Affiliations:** 1 Key Laboratory of Deep Underground Science and Engineering (Ministry of Education), College of Architecture and Environment, Sichuan University, Chengdu 610065, China; 2 Failure Mechanics and Engineering Disaster Prevention and Mitigation, Key Laboratory of Sichuan Province, Sichuan University, Chengdu 610065, China; 3 Sichuan Huashi Green Homeland Building Material Co., Ltd., Chengdu 610081, China

**Keywords:** sewage sludge ash, geopolymer, fluidity, setting time, compressive strength, comprehensive evaluation

## Abstract

The activity of sewage sludge ash (SSA) is not high; ground granulated blast slag (GGBS) has a high calcium oxide content that can accelerate polymerization rates and exhibit better mechanical performance. In order to improve the engineering application of SSA–GGBS geopolymer, it is necessary to conduct a comprehensive evaluation of its performance and benefits. In this study, the fresh properties, mechanical performance and benefits of geopolymer mortar with different SSA/GGBS, modulus and Na_2_O contents were studied. Taking the economic and environmental benefits, working performance and mechanical performance of mortar as evaluation indexes, the entropy weight TOPSIS (Technique for Order Performance by Similarity to Ideal Solution) comprehensive evaluation method is used to evaluate the geopolymer mortar with different proportions. The results show that as SSA/GGBS increases, the workability of mortar decreases, the setting time first increases and then decreases, and the compressive strength and flexural strength decrease. By appropriately increasing the modulus, the workability of the mortar decreases and more silicates are introduced, resulting in increased strength in the later stage. By appropriately increasing the Na_2_O content, the volcanic ash activity of SSA and GGBS is better stimulated, the polymerization reaction is accelerated, and the early strength increases. The highest $I_{c}$ (integrated cost index, $\frac{C_{t}}{f_{c28}})$ of geopolymer mortar is 33.95 CNY/m^3^/MPa, and the lowest is 16.21 CNY/m^3^/MPa, which is at least 41.57% higher than that of ordinary Portland cement (OPC). The minimum $I_{e}$ (embodied CO_2_ index, $\frac{E_{c}}{f_{c28}})$ is 6.24 kg/m^3^/MPa, rising up to 14.15 kg/m^3^/MPa, which is at least 21.39% lower than that of OPC. The optimal mix ratio is a water–cement ratio of 0.4, a cement–sand ratio of 1.0, SSA/GGBS of 2/8, a modulus content of 1.4, and an Na_2_O content of 10%.

## 1. Introduction

With the rapid improvement of living standards and the continuous population increase of, the production of sewage sludge worldwide is also increasing. Incineration of sewage sludge can considerably reduce the mass and volume of sewage sludge, which makes further management easier and leads to increasing production of incinerated sewage sludge ash (SSA) [1]. At the same time, the manufacturing process of cement accounts for about 5–8% of total CO_2_ emissions worldwide [2], so it is very necessary to use green cementing materials instead of cement. Geopolymers are prepared by excitation of solid precursor in an alkaline environment. The precursors are usually silicon-rich (Si) and aluminum-rich (Al) ground granulated blast slag (GGBS), fly ash, and metakaolin [3,4,5]. Compared to ordinary Portland cement, it is a low-carbon and sustainable cement material for the construction industry [6]. In addition to environmental benefits, geopolymers also have relatively good mechanical properties [7]. Studies have shown that higher incineration temperatures (800 °C) can change the chemical composition of SSA (increasing the amorphous properties) [8], resulting in a certain level of pozzolanic activity of SSA. This has led to the hypothesis that the use of SSA to produce an environmentally friendly geopolymer cementing material can both dispose of the increasing volume of SSA and reduce the production of cement. In view of this, some researchers have studied the application of SSA in geopolymers.

Research points out that the Si and Al are mostly in amorphous form in SSA, which improves the degree of geopolymerization reaction as well as the physical and chemical properties of geopolymer [9]. Furthermore, due to the unique 3D gel network structure of geopolymers, it is easy to solidify heavy metal ions. The use of SSA in the preparation of geopolymer can greatly reduce the pollution risk of heavy metal leaching [10,11,12,13]. Researchers studying geopolymer prepared from a mixture of SSA and other precursors including metakaolin, rice husk ash, fly ash and GGBS have mainly focused on the effects of raw material proportions and alkali activator composition on mechanical properties [9,14,15,16,17,18,19,20,21], while there is a lack of emphasis on performance, cost and carbon emissions, which are all important factors considered in practical applications. In addition, commercialization of geopolymers faces a challenge, as some silico-aluminate cannot be used in geopolymers, resulting in inconsistent cost and performance of geopolymers [22].

Therefore, future research should focus on whether the use of SSA in geopolymers is environmentally and economically feasible. In order to increase the possibility of application of SSA in geopolymers, it is necessary to comprehensively consider its economic benefits, environmental impacts, working performance and mechanical properties and select an optimal implementation scheme to maximize the benefits. In this study, SSA and GGBS are used as the raw materials to produce geopolymer mortar. Firstly, the effects of SSA/GGBS, modulus and Na_2_O content on the workability, setting time, compressive strength, flexural strength, cost and carbon emission of geopolymer mortar were studied, then the best ratio is obtained through comprehensive evaluation by the entropy weight TOPSIS (Technique for Order Performance by Similarity to Ideal Solution) method, which contributes to the practical application of SSA in geopolymer.

## 2. Materials and Methods

### 2.1. Raw Materials

The liquid alkali activator is a mixture of NaOH, Na_2_SiO_3_ and addition water in a certain proportion. The main indicators of Na_2_SiO_3_ are shown in Table 1, with a modulus of 3.13. NaOH is a solid particle.

A mixture of SSA and GGBS was used as the precursor. The SSA was obtained from an incinerator at a local sludge treatment plant and was incinerated at 850 °C, and the GGBS was commercially available S95-grade ore powder. Neither was mechanically ground with a ball mill in the laboratory. The chemical compositions of SSA and GGBS are shown in Table 2. The main chemical component of SSA is SiO_2_, with a content of up to 38.38%, followed by Al_2_O_3_ and P_2_O_5_, with contents of 18. 45% and 14.15%, respectively. The main components of GGBS are CaO, SiO_2_, and Al_2_O_3_, with contents of 33.49%, 26.39%, and 24.06%, respectively.

**Figure 1:**
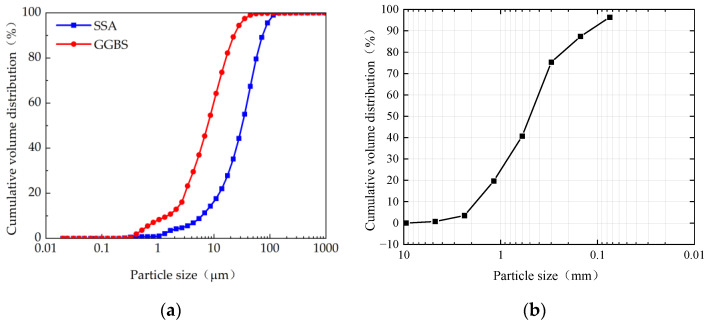
Particle size distribution of raw materials: (**a**) SSA and GGBS; (**b**) sand.

**Figure 2:**
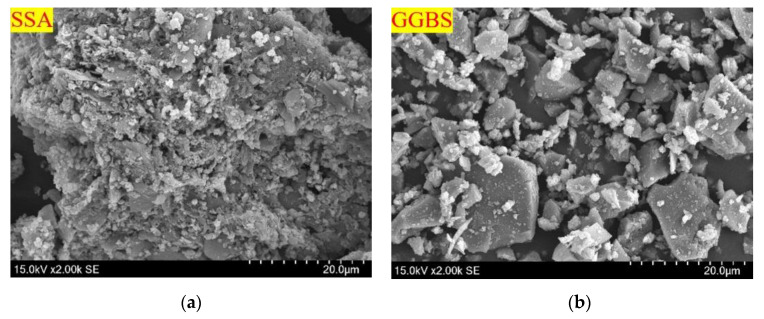
Micromorphology of SSA and GGBS: (**a**) SSA; (**b**) GGBS.

**Figure 3:**
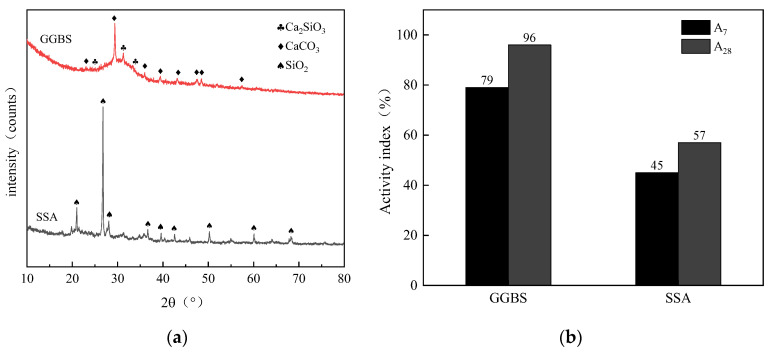
XRD and Activity of SSA and GGBS: (**a**) XRD; (**b**) activity.

The particle size distribution was measured for the GGBS and SSA by laser size diffraction, as shown in Figure 1. The average grain sizes of the SSA and GGBS are 36.65 μm and 13.44 μm, and the particle sizes are mainly distributed between 1.0–120 μm and 0.2–40 μm, respectively, indicating that the GGBS is much finer and the particle size distribution range is more concentrated than the SSA. The sand is medium sand with a fineness modulus of 2.25. The microstructure of SSA and GGBS are shown in Figure 2. The shape of SSA particles is irregular, with a rough surface and abundant pores. This structure increases its specific surface area and makes it easy to absorb water during stirring molding. The particles of GGBS are mainly blocky and have a smooth surface. Under the same magnification, particles of SSA are significantly larger than of GGBS. The mineralogy of the SSA and GGBS was determined by X-ray powder diffractometer (XRD), and the activity index of SSA and GGBS is indirectly measured by using the strength index method for to the ground granulated blast slag powder used in cement, mortar and concrete (GB/T 18046-2017), as shown in Figure 3. It is clear that SSA and GGBS consist of mainly amorphous humps characterized by baseline deviation between 25° and 35° (2θ), with a small calcite crystal peak in between, indicating that most of the minerals are in amorphous form. Figure 3a also shows that the SSA and GGBS have high crystallinity and contain certain crystalline minerals. Quartz (SiO_2_) is the most abundant mineral in SSA, and the crystal phases are mainly calcium silicate (Ca_2_SiO_3_) and calcite (CaCO_3_) in GGBS. The 7d and 28d activity indexes of GGBS are 79% and 95.77%, respectively, belonging to the S95 level. The 7d and 28d activity index of SSA are 45% and 57%, respectively. Compared to SSA, the activity index of GGBS is 75.56% higher at 7d, and 68.42% higher at 28d, indicating that the volcanic ash activity of GGBS is greater than that of SSA.

### 2.2. Mix Proportion

As there are many factors affecting the performance of geopolymer mortar, the water-to-binder ratio of 0.4 and sand-to-binder ratio of 1.0 remained unchanged. Note that the water content included the water in liquid alkali activator. The binder included the precursor and the solid components of Na_2_SiO_3_ (SiO_2_ and Na_2_O) and NaOH (Na_2_O). The preliminary experiment determined the SSA/GGB level (1/9–5/5), the modulus level (1.0–1.6) and the Na_2_O content level (6–12%).

Eleven mix proportions of SSA-GGBS geopolymer mortar were designed, as shown in Table 3. The SSA/GGBS is the mixture of SSA and GGBS by mass ratio. The modulus is the molar ratio of SiO_2_ to Na_2_O in the liquid alkali activator, and the Na_2_O content is the percentage of Na_2_O in the liquid alkali activator compared to the binder.

### 2.3. Test Method

**Figure 4:**
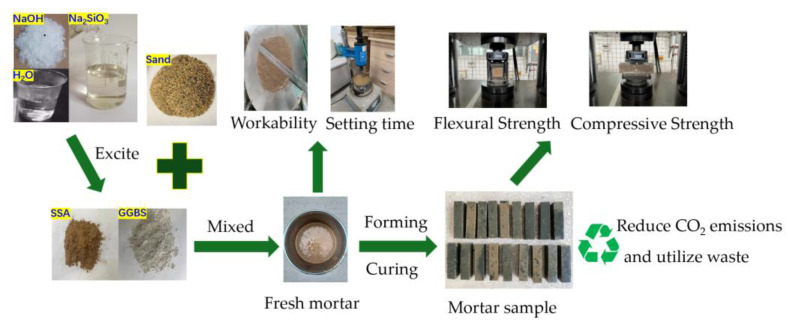
The preparation process of geopolymer mortar.

The preparation process of geopolymer mortar is shown in Figure 4. Firstly, the liquid alkali activator is prepared by mixing the Na_2_SiO_3_, NaOH and water in prescribed proportions; after mixing well, the solution was sealed to prevent evaporation and cooled to room temperature. The SSA and GGBS were placed in the mortar mixer in the laboratory and stirred for 1 min, then the alkali activator was poured into the mixer for 2 min, and then the river sand was poured into the mixer for 2 min to obtain the fresh mortar. The fluidity test of fresh mortar shall refer to the Method for Determining the Fluidity of Cement Mortar (GB/T2419-2005). The setting time test of fresh mortar shall refer to the Basic Performance Testing Methods for Building Mortars (JGJ/T70-2009). The fresh mortar was poured into a PVA mold with a size of 40 × 40 × 160 mm and vibrated for 30 s on a shaker to eliminate bubbles. The mortar was placed in a standard curing oven with a temperature of 20 ± 2 °C and a humidity of 95% until the test time. The flexural and compressive strength test of the geopolymer mortar shall refer to the Test Method for Strength of Cement Mortar (GB/T17671-2021). After testing the compressive strength at 28 days, part of the crushed samples was dried for scanning electron microscopy (SEM) analyses conducted with SU8020 to obtain the microstructure morphology of the geopolymer mortar.

## 3. Results and Discussion

### 3.1. Fresh Performance of Geopolymer Mortar

#### 3.1.1. Workability

**Figure 5:**
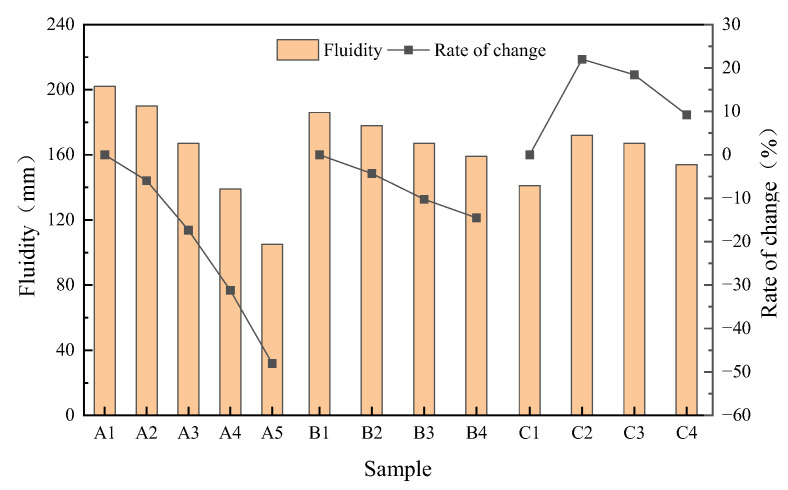
Fluidity of geopolymer mortar.

The fluidity of the geopolymer mortar is shown in Figure 5. The maximum fluidity of A1 is 202 mm, and the minimum of A5 is only 105 mm. As SSA/GGBS increased from 1/9 to 5/5, mortar fluidity decreased by 5.94%, 17.33%, 31.19% and 48.02%, respectively. As is shown in Figure 2, SSA has a rough surface structure, irregular particle shape and porous microstructure [23,24,25], which means that the use of SSA as a concrete filler will increase the water absorption of concrete by 8–20% [26,27]. Therefore, the more SSA used in the mixing process of geopolymer mortar, the more alkali activating liquid is absorbed, and the lower the fluidity. Yan [28] also pointed out that after adding dry wastepaper sludge to geopolymer mortar, the wastepaper sludge absorbed a large amount of water in the mortar mixture, resulting in a significant decrease in mortar fluidity. As the modulus increased from 1.0 to 1.6, the fluidity of mortar decreased by 4.30%, 10.22% and 24.19%, respectively. Due to the high viscosity of the Na_2_SiO_3_ solution [29], the higher the modulus, the higher the viscosity of the solution, and the corresponding viscosity of the mortar will also increase, so the fluidity of the mortar will decrease. As the Na_2_O content increased from 6% to 12%, the fluidity of the mortar increased by 21.99%, 18.44% and 9.22%, respectively. This is because the alkalinity of the system can be improved by properly increasing the Na_2_O content, which is conducive to the dissociation of the glass body at the early stage of hydration, thus reducing the internal friction between the particles and improving the fluidity; however, excessive alkali content will inhibit the reaction and reduce the fluidity [30].

#### 3.1.2. Setting Time

**Figure 6:**
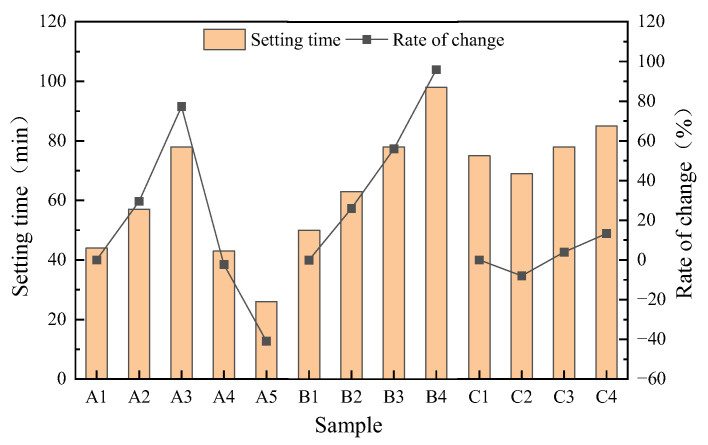
Setting time of geopolymer mortar.

The setting time of geopolymer mortar is shown in Figure 6. The maximum setting time of B4 is 98 min, and the minimum of A5 is only 26 min. As SSA/GGBS increased, the setting time of mortar first increased and then decreased. SSA/GGBS increases, the calcium content in the system decreases, and the absorption of alkaline activators by SSA increases. As is shown in Figure 3b, due to the lower activity of SSA compared to GGBS, the dissolution of calcium in GGBS is delayed, the rate of geopolymer polymerization reaction slows down, and the formation time of hydration products is prolonged, resulting in an extension of setting time. In addition, it was found that the setting time of geopolymer mortar was reduced by increasing the proportion of CaO in the raw material [31]; as is shown in Table 2, the content of CaO in SSA is much lower than that of GGBS, so the increase in SSA content will prolong the setting time of the mortar. However, when the amount of SSA added is too high (SSA/GGBS of 4/6) due to the absorption of a large amount of water by the SSA during the mixing process, the viscosity of the geopolymer mortar is also too high, resulting in a loss of plasticity and shortened setting time. As modulus increases from 1.0 to 1.6, the setting time is extended by 26%, 56% and 96%, respectively. The number of (SiO_4_)^4−^ monomers in the solution decreases and the number of polymers increases with the increase in the modulus, hindering the dissolution and condensation of solid precursors [32], which has a negative impact on the hydration process of the system, so the setting time is prolonged. As Na_2_O content increases, the setting time of mortar decreased first and then increased. The change process of zeta potential in the system follows the same pattern as the condensation time [33]. The (SiO_4_)^4−^ and (AlO_4_)^5−^ released by the decomposition of monomer materials in the geopolymer system are negative charges, and the Na^+^ introduced by Na_2_O is positive charges; together they affect the zeta potential within the system. By appropriately increasing the Na_2_O content, positive and negative ions attract each other and the polymerization speed accelerates, so the setting time decreases. Continuing to increase the Na_2_O content will lead to an increase in the amount of Na^+^ introduced into the system, so the repulsion between ions increases, polymerization reactions are inhibited, and the setting time increases.

### 3.2. Mechanical Performance of Geopolymer Mortar

#### 3.2.1. Compressive Strength and Flexural Strength

**Figure 7:**
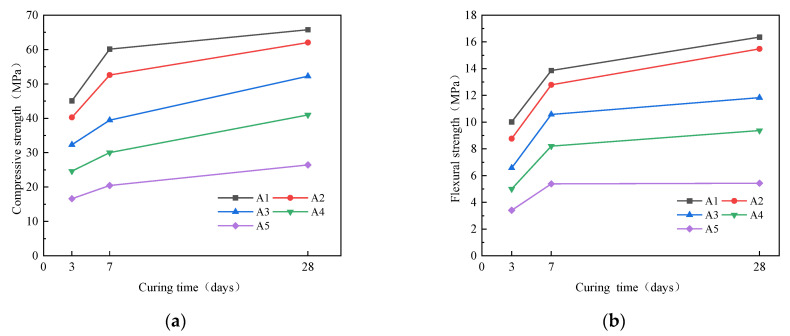
Effect of SSA/GGBS on strength of mortar: (**a**) Compressive; (**b**) Flexural.

The effect of SSA/GGBS on strength of geopolymer mortar is shown in Figure 7. As SSA/GGBS increased, the compressive and flexural strength both decreased. The compressive strength of A1 group at each age is the highest, reaching 45.09 MPa at 3d, 60.12 MPa at 7d and 65.77 MPa at 28d. The compressive strength of A5 at each age is the lowest, reaching 16.61 MPa at 3d, 20.46 MPa at 7d and 26.45 MPa at 28d, constituting decreases by 63.16%, 65.97%, and 59.78%, respectively. The flexural strength of A1 is the highest, reaching 10.02 MPa at 3d, 13.86 MPa at 7d and 16.36 MPa at 28d. The flexural strength of A5 is the lowest, reaching 3.41 MPa at 3d, 5.38 MPa at 7d and 5.43 MPa at 28d, constituting decreases by 65.97%, 61.16%, and 66.81%, respectively. The reaction of the geopolymer polymerization process is as in Equations (1) and (2) below [34]. It can be seen that the calcium content has a great impact on the compressive strength. The calcium content in SSA is lower than that of GGBS, so when SSA/GGBS increases, the calcium content in the system decreases, the content of C-(A)-S-H gel generated decreases, and the strength decreases. Because the contribution of GGBS to strength plays a dominant role, and the activity of GGBS is higher than that of SSA, the polymerization rate in the early stage is fast, and its strength develops slowly from 7d to 28d correspondingly. The content of GGBS in A1 accounts for 90%, and the compressive strength at 7d is very close to that at 28d.

(1) $${{Na}^{+}+SiO}_{2}{\left(OH\right)}_{2}^{2-}/Si{\left(OH\right)}_{3}^{1-}+Al{\left(OH\right)}_{4}^{-}\to N-A-S-H\;\mathrm{gel}$$

(2) $${Ca}^{2+}{+SiO}_{2}{\left(OH\right)}_{2}^{2-}/Si{\left(OH\right)}_{3}^{1-}+Al{\left(OH\right)}_{4}^{-}\to C-(A)-S-H\;\mathrm{gel}$$

**Figure 8:**
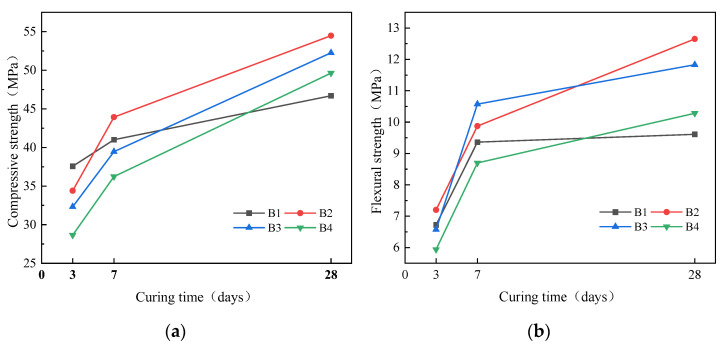
Effect of modulus on strength of mortar: (**a**) compressive; (**b**) flexural.

The effect of modulus on strength of geopolymer mortar is shown in Figure 8. As modulus increased, the compressive strength at 3d decreased. Compared with B1, the compressive strength of B4 at 3d decreased by 24.19%. In contrast, the compressive strength at 28d first increased and then decreased. Compared with B1, the compressive strength of B2, B3 and B4 at 28d increased 16.66%, 11.93% and 6.30%, respectively. The flexural strength at 28d first increases and then decreases as the modulus increases. Compared with B1, the flexural strength of B2, B3 and B4 at 28d increased by 31.63%, 23.10% and 6.97%, respectively. The effect of modulus on the strength of geopolymer mortar varies at different curing ages. As modulus increases, the alkalinity of the alkali solution decreases, which will affect the dissolution of silicon aluminum monomers in early polymerization reaction, resulting in a decrease in the strength at 3d. At the later stage of polymerization, increasing the modulus can introduce more silicates, more aluminate silicate gel can be generated, so the strength at 28d increases. However, when the modulus content is too high, due to the introduction of too much soluble SiO_2_, it may cover the surface of unreacted SSA and GGBS particles during the polymerization reaction process, reducing the degree of polymerization reaction, resulting in a decrease in the strength at 28d.

**Figure 9:**
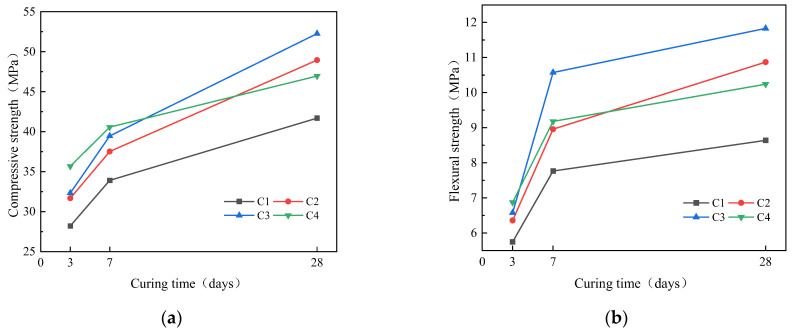
Effect of Na_2_O content on strength of mortar: (**a**) compressive; (**b**) flexural.

The effect of Na_2_O content on the strength of geopolymer mortar is shown in Figure 9. As Na_2_O content increased, the compressive strength at 3d and 7d increased. Compared with C1, the compressive strength of C4 at 3d and 7d increased by 26.44% and 19.56%. While the compressive strength at 28d first increased and then decreased. Compared with C1, the compressive strength of C2, C3 and C4 at 28d increased by 17.41%, 25.35% and 12.61%, respectively; The flexural strength at 3d increased as Na_2_O content increased. Compared with C1, the flexural strength of C4 at 3d increased by 19.56%. The flexural strength at 7d and 28d first increases and then decreases. Compared with C1, the flexural strength of C2, C3 and C4 at 7d increased by 15.32%, 36.12% and 18.20%, while the flexural strength of C2, C3 and C4 at 28d increased by 2.45%, 36.92% and 18.52%, respectively. The reaction of the polymer depolymerization process is as in Equations (3)–(5) below [35]. Silica aluminum oxide is hydrolyzed in an alkaline environment to form a geopolymer gel network. Therefore, with the increase in Na_2_O content, the OH^−^ concentration increases and more active silicon aluminum monomers are dissolved in SSA and GGBS during the early stage of the reaction. Furthermore, the increase in Na_2_O content also introduces additional Na^+^, which is conducive to the formation of C-A-S-H gel and N-A-S-H gel [36], so the strength at 3d is improved. However, excessive OH^−^ will cause the condensation reaction of the geopolymer to occur faster and earlier, leading to an immature structure [37]; thus, strength at 28d decreases.

(3) $${Al_{2}O_{3}+3H}_{2}O+2OH^{-}\to 2{\left[Al{\left(OH\right)}_{4}\right]}^{-}$$

(4) $${SiO}_{2}+H_{2}O+{OH}^{-}\to {\left[SiO{\left(OH\right)}_{3}\right]}^{-}$$

(5) $${SiO}_{2}+2{OH}^{-}\to {\left[SiO_{2}{\left(OH\right)}_{2}\right]}^{2-}$$

**Figure 10:**
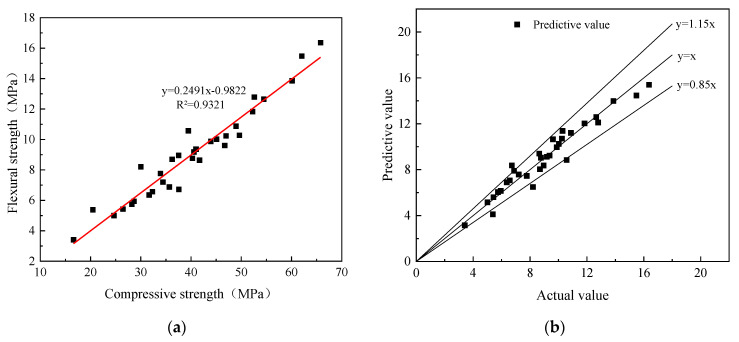
Relationship between compressive strength and flexural strength: (**a**) fitting results; (**b**) error range.

The linear fitting results of the compressive strength and flexural strength of geopolymer mortar are shown in Figure 10. According to the Equation (6), the flexural strength is basically 1/4 of the compressive strength, which is different from cement mortar. The flexural strength in cement mortar is generally 1/10–1/5 of the compressive strength, indicating that geopolymer mortar has good toughness. As shown in Figure 10, the predictive values calculated by Equation (6) are close to the actual values and the error range is basically within 15%, indicating that the fitting formula has good applicability.

(6) $$f_{t}=0.2491f_{c}-0.9822,$$

#### 3.2.2. SEM Analysis

**Figure 11:**
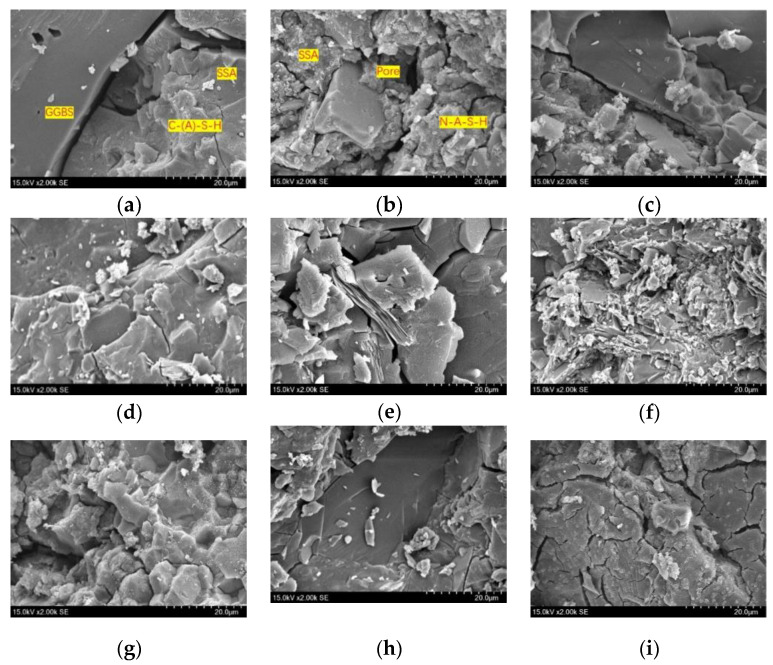
Micromorphology of geopolymer mortar: (**a**) A1; (**b**) A5; (**c**) B1; (**d**) B2; (**e**) B3/C3; (**f**) B4; (**g**) C1; (**h**) C2; (**i**) C4.

The microstructure of the geopolymer mortar after 28 days of curing is shown in Figure 11. The geopolymer matrix of A1 is denser than that of A5. The main polymerization products of GGBS and SSA are C-A-S-H gel and N-A-S-H gel, respectively. Compared with N-A-S-H gel, C-A-S-H gel has a more compact structure [38,39]. As SSA/GGBS increases, the ratio of C-A-S-H/N-A-S-H in the gel network decreases, and the gel network becomes sparse, leading to a decline in the strength of the geopolymer mortar.

The geopolymer matrix of B2 is denser than that of B1. This is because increasing the modulus can increase the indeterminate SiO_2_ in the system, which is conducive to the formation of geopolymer gel. However, with the further increase in the modulus, the surface of the geopolymer matrix of B3 and B4 is covered with a large amount of aluminate gel, which hinders the further hydration of SSA and GGBS particles, leading to a decrease in strength.

Due to the low alkalinity, the polymerization reaction of C1 is insufficient, and there are many voids in the geopolymer matrix. The degree of polymerization of C2 increases, most of the voids are filled with gel, so the strength is higher. Due to the high alkalinity, the polymerization reaction speed of C4 is too fast and there were many cracks in the later stage of the structure, resulting in a decrease in strength.

### 3.3. Benefits of Geopolymer Mortar

The current prices and CO_2_ emissions of raw materials used in geopolymer mortar are shown in Table 4. Due to the fact that SSA is obtained as recycled solid waste from local sewage sludge treatment plants, its cost is zero. The carbon emissions of H_2_O and sand come from an industry standard in China (T/CBMF 27-2018). 

In order to evaluate the economic efficiency and carbon footprint of geopolymers in this study, an integrated cost index $I_{c}$ (¥/m^3^/MPa) and an embodied CO_2_ index $I_{e}$ (kg/m^3^/MPa) were adopted as Equation (7) [42] and Equation (8) [43] below.

(7) $$I_{c}=\frac{C_{t}}{f_{c28},}$$

(8) $$I_{e}=\frac{E_{c}}{f_{c28}}$$

where $I_{c}$ is the integrated cost index, $I_{e}$ is the embodied CO_2_ index, $C_{t}$ (CNY/m^3^) is the cost of one cubic meter of geopolymer, embodied CO_2_ (kgCO_2_/m^3^) is the total CO_2_ emission of one cubic meter of geopolymer, and $f_{c28}$ (MPa) is the compressive strength at 28d of geopolymer.

#### 3.3.1. Cost

**Figure 12:**
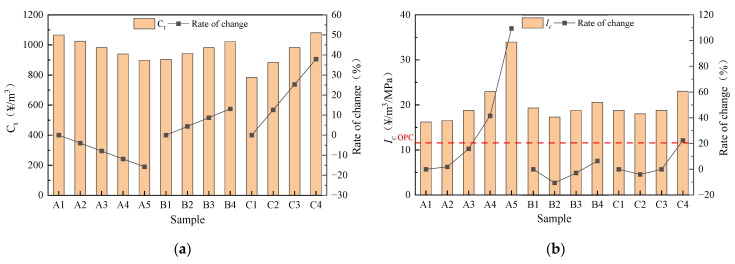
Cost of geopolymer mortar: (**a**) $C_{t}$; (**b**) $I_{c}$.

The cost and $I_{c}$ of geopolymer mortar with different mix ratios are shown in Figure 12. As SSA/GGBS decreases and modulus and Na_2_O content increase, the cost increases. The maximum cost of group A1 was 1066.28 CNY/m^3^, while the minimum cost of Group C1 was 783.80 CNY/m^3^. The impact of Na_2_O content on cost is most significant; this is because the cost of NaOH is the most expensive. The $I_{c}$ shows a trend of first decreasing and then increasing with the increase in modulus and Na_2_O content, but all are around 20 CNY/m^3^/MPa. SSA/GGBS increases, the $I_{c}$ increases. The minimum $I_{c}$ of Group A1 is 16.21 CNY/m^3^/MPa and the maximum $I_{c}$ of Group A5 is 33.95 CNY/m^3^/MPa, with an increase of 109.47%. The $I_{c}$ of OPC (Ordinarily Portland cement) is 11.45 CNY/m^3^/MPa [44], which means that the cost of geopolymer mortar is at least 41.57% higher than that of OPC under the same strength. This is mainly because the geopolymer requires the use of alkaline activators to excite, while the cost of sodium hydroxide and sodium silicate is relatively expensive. In addition, liquid activators are corrosive and difficult to store, transport and handle, which affects the large-scale production of geopolymers. Therefore, safe and convenient solid activators can be developed to further reduce the acquisition cost of raw materials, so that the production of geopolymers can be scaled up and commercialized to make up for the shortage of geopolymers for engineering applications.

#### 3.3.2. Carbon Emission

**Figure 13:**
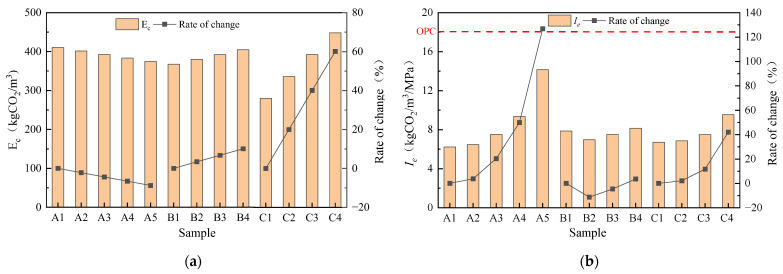
Carbon emission of geopolymer mortar: (**a**) $E_{c}$; (**b**) $I_{e}$.

The carbon emissions and $I_{e}$ of geopolymer mortar with different mix ratios are shown in Figure 13. As SSA/GGBS increases and modulus and Na_2_O contents decrease, the carbon emissions of geopolymer mortar decrease. The maximum carbon emissions of Group C4 were 410.40 kgCO_2_/m^3^, while the minimum carbon emissions of Group C1 were 280.03 kgCO_2_/m^3^, an increase of 60.13%. The Na_2_O content has the most significant impact on the carbon emissions of geopolymer mortar, as the increase in Na_2_O content led to the most significant increase in NaOH, which has the highest carbon emission factor. The $I_{e}$ values are all around 8 kgCO_2_/m^3^/MPa with different modulus and Na_2_O contents. The increase in SSA/GGBS has to some extent reduced carbon emissions. However, due to the decrease in strength, it actually leads to an increase in $I_{e}$. The minimum $I_{e}$ of Group A1 is 6.24 kgCO_2_/m^3^/MPa, while the maximum $I_{e}$ of group A5 is 14.15 kgCO_2_/m^3^/MPa, an increase of 126.79%. The $I_{e}$ of OPC is 18 kgCO_2_/m^3^/MPa [43] and the carbon emission of geopolymer mortar is at least 21.39% lower than that of OPC under the same strength, indicating that the geopolymer has superior environmental friendliness compared to OPC.

### 3.4. Comprehensive Evaluation of Performance and Benefits

The TOPSIS (Technique for Order Performance by Similarity to Ideal Solution) method is a sorting method that approximates ideal solutions [45], which can determine the degree of closeness to the optimal solution in the sample data. The entropy weight TOPSIS method is a combination of the entropy weight method and the TOPSIS method. By using entropy to determine weights, it eliminates the shortcomings of subjective weights [46].

#### 3.4.1. Indicator Weight

Considering comprehensively the benefits, workability, and mechanical properties of geopolymer mortar, a total of 10 evaluation indicators were selected for the preparation cost. In terms of mortar efficiency, the indicator attribute is negative, which means that the lower the preparation cost and carbon emissions, the better the overall performance, as shown in Table 5.

The process of calculating the weight of evaluation indicators using the entropy weight method is as follows:

(1) Build evaluation matrix $R_{X}$. $X_{ij}$ is the raw data corresponding to the *j*th evaluation indicators of the *i*th evaluation object (*i* = 1, 2, …, *m*; *j* = 1, 2, …, *n*). The raw data are shown in Table 6.

(9) $$R_{X}=\left[\begin{array}{ccc} X_{11} & \cdots & X_{1n}\\ \vdots & X_{ij} & \vdots \\ X_{m1} & \cdots & X_{mn} \end{array}\right]$$

(2) Indicator standardization $R_{Y}$. Standardize raw data based on indicator attributes.

For positive indicators:

(10) $$Y_{ij}=\frac{X_{ij}-\min\left(X_{1j},\cdots ,X_{mj}\right)}{\max\left(X_{1j},\cdots ,X_{mj}\right)-\min\left(X_{1j},\cdots X_{mj}\right)}$$

For negative indicators:

(11) $$Y_{ij}=\frac{\max\left(X_{1j},\cdots ,X_{mj}\right)-X_{ij}}{\max\left(X_{1j},\cdots ,X_{mj}\right)-\min\left(X_{1j},\cdots X_{mj}\right)}$$

(3) Calculate the weight of evaluation indicators:

(12) $$p_{ij}=\frac{y_{ij}}{\sum_{j=1}^{m}y_{ij}}$$

(13) $$e_{i}=-\frac{1}{\ln n}\sum_{j=1}^{m}P_{ij}lnP_{ij}$$

(14) $$W_{i}=\frac{1-e_{i}}{\sum_{i=1}^{n}\left(1-e_{i}\right)}$$

Normalize the indicators sequentially through Equations (10)–(14), determine the entropy value, and calculate the weight. The results are shown in the table below in Table 7.

It can be seen from Table 7 that in the three dimensions of the first level indicators, the proportion weights of the benefits, fresh performance, and mechanical performance of geopolymer mortar are 26.62%, 18.69%, and 54.69%, respectively. This indicates that mechanical performance is the most important factor affecting the comprehensive performance of geopolymer mortar, which is consistent with the selection basis of materials in practical engineering applications.

#### 3.4.2. Evaluation Results

After obtaining the weights of each evaluation index through the entropy weight method, the TOPSIS method is used to comprehensively evaluate the performance of geopolymer mortar with different mix ratios. The process is as follows:

(1) Building a Weighted Normalization Matrix $R_{V}$. $V_{ij}$ = $W_{i}$ × $X_{ij}$:

(15) $$V=\left[\begin{array}{ccc} V_{11} & \cdots & V_{1n}\\ \vdots & V_{ij} & \vdots \\ V_{m1} & \cdots & V_{nm} \end{array}\right]$$

(2) Determine positive and negative ideal solutions and Euclidean distance:

The minimum value of the negative index and the maximum value of the positive index constitute the set of positive ideal solutions; The maximum value of the negative index and the minimum value of the positive index constitute the set of negative ideal solutions. Use the number set to express its positive ideal solution $V^{+}$, and negative ideal solution $V^{-}$:

(16) $$V^{+}=\left\{V_{1}^{+},\cdots ,V_{j}^{+},\cdots ,V_{n}^{+}\right\}$$

(17) $$V^{-}=\left\{V_{1}^{-},\cdots ,V_{j}^{-},\cdots ,V_{n}^{-}\right\}$$

(3) After the positive and negative ideal solutions are determined, calculating the positive ideal solution distance $d^{+}$, the negative ideal solution distance $d^{-}$ and the relative fitness C of the evaluation object respectively through Equations (18)–(20). The comprehensive evaluation results are shown in Table 8.

(18) $$d_{i}^{+}=\sqrt{\sum_{j=1}^{n}{\left(V_{ij}-V_{j}^{+}\right)}^{2}}$$

(19) $$d_{i}^{-}=\sqrt{\sum_{j=1}^{n}{\left(V_{ij}-V_{j}^{-}\right)}^{2}}$$

(20) $$C_{i}=\frac{d_{i}^{-}}{d_{i}^{-}+d_{i}^{+}}$$

According to the principle of the greater the relative superiority, the better, the best mix ratio is A2. That is to say, the mortar with SSA/GGBS of 2/8, a modulus of 1.4 and Na_2_O content of 10% has the best comprehensive performance.

## 4. Conclusions

In order to further realize the resource utilization of SSA, this study prepared geopolymer mortar through the synergistic preparation of SSSA and GGBS. The effects of SSA/GGBS, modulus and Na_2_O content on the fresh performance, mechanical performance, economic benefits, and environmental benefits of SSA-GGBS geopolymer mortar were studied. The entropy-weighted TOPSIS method was used to comprehensively evaluate the performance and benefits of geopolymer mortar and obtain the optimal mix ratio, and the following conclusions were obtained:

1. As SSA/GGBS increases, the irregular particle morphology and porous surface structure of SSA make it highly susceptible to moisture absorption, resulting in a decrease in fluidity. The activity of SSA is lower compared to GGBS, the polymerization reaction speed of geopolymer is slowed down, the setting time of mortar increases, the CaO content in SSA is less, the gel network generated by geopolymer polymerization becomes sparse, and the mortar strength decreases.
2. As modulus increases, the viscosity of solution increases and the fluidity decreases. Due to the introduction of more silicates, the later strength of mortar is improved. As Na_2_O content increases, the pozzolanic activity of SSA and GGBS can be better stimulated, the setting time is reduced, and the early strength of mortar is improved due to the accelerated polymerization reaction.
3. Because alkali activators are expensive and have high carbon emissions, the maximum $I_{c}$ of geopolymer mortar is 33.95 CNY/m^3^/MPa, and the minimum is 16.21CNY/m^3^/MPa, which is at least 41.57% higher than that of OPC. The minimum $I_{e}$ is 6.24 kg/m^3^/MPa, while the maximum is 14.15 kg/m^3^/MPa, which is at least 21.39% lower than that of OPC.
4. The weights of benefit, fresh performance, and mechanical performance in the comprehensive evaluation model are 26.62%, 18.69% and 54.69%. The optimal mix ratio is a water–cement ratio of 0.4, a cement–sand ratio of 1.0, SSA/GGBS of 2/8, a modulus of 1.4, and an Na_2_O content of 10%.

## Figures and Tables

**Table 1:** Main indicators of Na_2_SiO_3_ (%).

Indicators	SiO_2_	Na_2_O	Solid Phase	Liquid Phase
Content	27.64	8.83	36.47	63.53

**Table 2:** Chemical composition of SSA and GGBS (%).

Materials	SSA	GGBS
SiO_2_	38.38	26.39
Al_2_O_3_	18.45	24.06
Fe_2_O_3_	8.16	0.73
CaO	4.69	33.49
MgO	3.15	7.53
Na_2_O	0.66	0.72
TiO_2_	1.04	1.41
P_2_O_5_	14.15	0.34

**Table 3:** Mix proportion of geopolymer mortar (kg·m^−3^).

Sample	SSA/GGBS	Modulus	Na_2_O Content	SSA	GGBS	Na_2_SiO_3_	NaOH	H_2_O	Sand
A1	1/9	1.4	10%	76.45	688.06	490.17	73.18	72.13	1000
A2	2/8	1.4	10%	152.90	611.61	490.17	73.18	72.13	1000
A3 (B3, C3)	3/7	1.4	10%	229.35	535.16	490.17	73.18	72.13	1000
A4	4/6	1.4	10%	305.81	458.71	490.17	73.18	72.13	1000
A5	5/5	1.4	10%	382.26	382.26	490.17	73.18	72.13	1000
B1	3/7	1.0	10%	240.97	562.26	350.12	89.14	157.51	1000
B2	3/7	1.2	10%	235.16	548.71	420.15	81.16	114.82	1000
B4	3/7	1.6	10%	223.55	521.61	560.20	65.21	29.43	1000
C1	3/7	1.4	6%	257.61	601.10	294.10	43.91	203.28	1000
C2	3/7	1.4	8%	243.48	568.13	392.14	58.55	137.70	1000
C4	3/7	1.4	12%	215.23	502.19	588.21	87.82	6.55	1000

**Table 4:** Cost and CO_2_ emission of materials.

Materials	Cost for One Ton (CNY)	CO_2_ Emission (kgCO_2_/m^3^)
SSA	0	0.025
GGBS	550	0.143 [40]
Na_2_SiO_3_	2000	0.387
NaOH	900	1.59 [41]
H_2_O	3.46	0.000148
Sand	100	0.00398

**Table 5:** Comprehensive evaluation indicators.

Primary Indicators	Secondary Indicators	Variable	Unit	Attribute
Benefits	Cost	X_11_	CNY/m^3^	-
	CO_2_ emission	X_12_	kgCO_2_/m^3^	-
Fresh performance	Fluidity	X_21_	mm	+
	Setting time	X_22_	min	+
Mechanical performance	Compressive strength at 28d	X_31_	MPa	+
	Flexural strength at 28d	X_32_	MPa	+
	Compressive strength at 7d	X_33_	MPa	+
	Flexural strength at 7d	X_34_	MPa	+
	Compressive strength at 3d	X_35_	MPa	+
	Flexural strength at 3d	X_36_	MPa	+

**Table 6:** Evaluation matrix Rx raw data.

Sample	X_11_	X_12_	X_21_	X_22_	X_31_	X_32_	X_33_	X_34_	X_35_	X_36_
A1	1066.28	410.40	202	44	45.09	60.12	65.77	10.02	13.86	16.36
A2	1024.21	401.37	190	57	40.26	52.59	62.04	8.76	12.79	15.48
A3	982.19	392.35	167	78	32.32	39.46	52.26	6.58	10.57	11.83
A4	940.11	383.33	139	43	24.65	30.02	40.98	5.00	8.20	9.37
A5	898.09	374.31	105	26	16.61	20.46	26.45	3.41	5.38	5.43
B1	903.10	367.59	186	50	37.56	41.00	46.69	6.72	9.36	9.61
B2	942.67	380.03	178	63	34.40	43.93	54.47	7.20	9.87	12.65
B4	1021.56	404.63	159	98	28.65	36.23	49.63	5.94	8.70	10.28
C1	783.80	280.03	141	75	28.21	33.90	41.69	5.75	7.77	8.64
C2	882.82	336.08	172	69	31.67	37.51	48.95	6.36	8.96	10.87
C4	1081.21	448.41	154	85	35.67	40.56	46.95	6.88	9.18	10.24

**Table 7:** Evaluation index weight.

Index	e	w	Total
X_11_	0.885	15.42%	26.62%
X_12_	0.916	11.20%	
X_21_	0.939	8.10%	18.69%
X_22_	0.921	10.59%	
X_31_	0.941	7.94%	54.69%
X_32_	0.929	9.57%	
X_33_	0.929	9.54%	
X_34_	0.928	9.68%	
X_35_	0.937	8.49%	
X_36_	0.929	9.47%	

**Table 8:** Ranking of comprehensive evaluation results.

Materials	$d^{+}$	$d^{-}$	C	Rank
A1	0.515597	0.80035	0.608193	2
A2	0.454622	0.706675	0.608522	1
A3	0.496177	0.539081	0.520722	7
A4	0.669104	0.345128	0.340285	10
A5	0.889749	0.283107	0.241382	11
B1	0.488341	0.548663	0.529084	6
B2	0.444428	0.576844	0.564829	3
B4	0.594678	0.500167	0.456838	8
C1	0.530474	0.625433	0.541075	5
C2	0.452038	0.567486	0.556619	4
C4	0.648903	0.489812	0.430145	9

## Data Availability

Data are contained within the article or are available on request from the corresponding author.
